# Pathogen Lists Do Not Tell Us What We Need to Do

**DOI:** 10.4269/ajtmh.19-0084

**Published:** 2019-03-18

**Authors:** David S. Fedson

## Abstract

Brett-Major and others remind us that pathogen lists for emerging infectious diseases aid in the development of tools that target specific pathogens (e.g., vaccines) and help attract financial support. These lists tell us what we need to have, not what we need to do. The authors call for more research on ways to prevent these diseases (e.g., platform technologies for vaccines) and mitigate disease impact. Vaccines and new treatments that target individual pathogens have many limitations. However, we might save lives by treating patients with inexpensive generic drugs that target common features of the host response to infection. Undertaking research on this approach to treatment is what we need to do.

## INTRODUCTION

In a recent perspective piece, Brett-Major, Racine, and Kobinger presented a critique of pathogen lists that focus on individual pathogens (mostly viruses) thought most likely to cause future epidemics or pandemics.^1^ These lists aid in the development of tools that target specific pathogens, attract the attention of funding organizations, and largely determine the organization of research. Brett-Major and others cite several examples of the disparities in research support for certain pathogens (e.g., Crimean–Congo hemorrhagic fever, Nipah and Rift Valley fever viruses versus Ebola and Zika viruses) and the low level of support for developing platform technologies that could be used to target multiple pathogens. Pathogen lists focus largely on traditional product development and, in consequence, lead to missed opportunities “to seek broadly applicable solutions to challenges in preventing disease and mitigating epidemics.”^1^ The authors argue that “We need less attention on what makes pathogens different and more on how they are alike in the ways that they cause outbreaks and impact communities,” and conclude “We should emphasize the question of what we need to do rather than what we need to have.”

## LIMITATIONS OF PATHOGEN LISTS

Winston Churchill said “It is no use saying ‘We are doing our best’. You have got to succeed in doing what is necessary.” Brett-Major and others are absolutely correct in saying that our focus should be on what we need to do, not what we need to have. Some outbreaks cause extensive morbidity but negligible mortality, so a better understanding of how they occur and affect communities is probably not urgently needed. Other outbreaks, however, are more important because they have high case fatality rates. For example, the ongoing Ebola outbreak in the Democratic Republic of the Congo has an overall case fatality rate of 59% and patients hospitalized with H7N9 influenza in China (most of whom were treated with antivirals) had case fatality rates of 40%.^2^ Thus, in my view, we do not need more research on the cause and community impact of these high-mortality outbreaks; instead, we need more research on how to save lives when these outbreaks occur.

## WILL PATHOGEN LISTS SAVE LIVES?

Brett-Major and others rightly challenge us to think differently. Much of their perspective piece is focused on what we need in the way of new vaccines (one of the co-authors is a leading investigator on Ebola vaccines^3^). The authors support developing new platform technologies that can be used to make different kinds of vaccines. They also urge us to improve regulatory pathways to get them licensed. Some of this work has already begun. The Coalition for Epidemic Preparedness Innovations is a generously funded global partnership between public, private, philanthropic, and civil society organizations working to accelerate the development of vaccines against emerging infectious diseases.^4^ Given the scale of these investments, these efforts might in the long run lead to new and efficacious vaccines. However, it is impossible to predict which pathogens will cause future outbreaks, so we do not really know which specific vaccines we will need to have. For the next influenza pandemic, we already know that for the foreseeable future, no one in the world will have access to pandemic vaccines for the first 6 months, a period during which it is estimated that almost 33 million people could die.^2^ Moreover, even if more research allows us to rapidly develop, register, and produce the specific vaccines we will need, developing countries in particular will still face huge logistical challenges in undertaking vaccination programs.

Advances in vaccine and antimicrobial technology mean that some emerging disease threats could be mitigated. For example, new influenza vaccines (including a “universal” influenza vaccine) might be shown to be efficacious. However, when the next pandemic virus emerges, they will not be of much help to a developing country like Bangladesh where people will not have timely access to supplies of these vaccines, will not be able to afford them, and because they do not use seasonal influenza vaccines, they will not have the human infrastructure to administer them. In a developing country faced with an Ebola-like epidemic, an efficacious vaccine that requires storage at below −60°C, costs tens of millions to produce and deliver, and is in short supply will probably not be an effective way to save lives. Moreover, even if investigational treatments for these diseases (e.g., new antivirals or monoclonal antibodies) are shown to be efficacious, they will be largely unavailable and/or unaffordable for most people, and some might even require parenteral administration. In short, the limitations of targeting individual pathogens (social, political, and economic, not just scientific) will be difficult to overcome.

## TREATING THE HOST RESPONSE TO INFECTION

For patients with an emerging infectious disease, saving lives might not depend on knowing the causative pathogen if the host response to this infection shares features common to the host response to other pathogens.^5^ More than a decade ago, the idea of treating the host response was proposed as a way to manage patients with pandemic influenza, and in 2014, it was suggested as a way to treat patients with Ebola in West Africa.^6^ A poorly documented treatment experience in Sierra Leone suggested that using a statin/angiotensin receptor blocker combination saved lives.

We already have important clues on how treatment that targets the host response (not the pathogen) might work. For example, improving tolerance to an infection might be more important than increasing resistance to the pathogen. Infections place enormous demands on energy metabolism, and the immunometabolic effects of drug treatment might improve survival.^6^ Evolution might help explain how host response treatment works.^7^

For pandemic influenza, an Ebola-like disease, and many other emerging infectious diseases, the only possibility for saving lives might be to use a treatment that is inexpensive, known to be safe, familiar to ordinary physicians, suitable for oral administration, and immediately available in any country with a basic health-care system.^6^ Many of the candidate drugs (e.g., statins,^8^ angiotensin receptor blockers, macrolides, and glucocorticoids) are produced as generics in developing countries and logistical systems for their delivery are already in place. These drugs (especially in combination) could be used in all countries on the first epidemic or pandemic day. To be effective, they will have to target common features of the host response to infections caused by all of these pathogens.^6^ The same approach might be used to treat patients with severe infections that occur every day (e.g., seasonal influenza, sepsis). In this way, generic drug treatment would be similar to oral rehydration solution—an inexpensive syndromic treatment for acute diarrheal diseases regardless of cause.^9^ There is even the possibility that these drugs might be used to help patients survive infections with antimicrobial-resistant bacteria.^10^

## WHAT WE NEED TO DO

Several publications in high-profile journals have reviewed the pathogenesis and treatment of influenza and Ebola and the lessons learned from the Ebola outbreak in West Africa.^6^ None of these articles mentioned host response treatment with inexpensive generic drugs. Influenza and Ebola scientists and health officials who support their work and count on their advice (including those at the WHO) have shown no interest in this idea. There is no guarantee this approach to treatment will work; we still need convincing evidence from clinical research to show that it does. Yet, if we care about saving lives in developing countries, we must urgently undertake research on treatments that target common features of the host response. This is what is necessary; this is what we need to do.
